# Evaluation of Jordan’s citizens’ awareness of the emerging Coronavirus (COVID-19) disease: A mixed analysis of the symptoms, transmission, and preventive measures

**DOI:** 10.1371/journal.pgph.0001041

**Published:** 2022-09-28

**Authors:** Inas Almazari, Roba Bdeir, Haneen A. Basheer

**Affiliations:** 1 Department of Clinical Pharmacy, Faculty of Pharmacy, Zarqa University, Zarqa, Jordan; 2 Faculty of Pharmacy, Jadara University, Irbid, Jordan; Tunisian Institute of Veterinary Research, TUNISIA

## Abstract

This study aimed to evaluate Jordan citizens’ awareness, knowledge, and practice concerning Coronavirus-19 (COVID-19) symptoms, routes of transmission, and preventive measures. An online self-administered questionnaire was filled out completely by participants (N = 328) from mainly four major cities in Jordan during the period beginning of May-end of September 2020. Participants’ main sources of knowledge about COVID-19 were the government websites (87.8%), social media (87.5%), and Television (TV) (81.1%). The majority of participants valued the drizzle of cough from infected individuals (96.3%), direct contact with contaminated surfaces (91.5%), and direct contact with infected individuals (84.5%) as the highest rates of the route of transmission. The highest rates chosen as symptoms of COVID-19 viral infections were high-grade fever (99.1%), troublesome breathing (96.6%), coughing (92.7%), headache (91.2%), and loss of smell and taste (80.8%). The majority of participants (>92%) strongly agreed on the behavioral protective measures such as no face touching, wearing a mask, the use of alcoholic hand disinfectants, and the need for self-isolation. This study showed that the Jordanian citizens were aware of the epidemiology of COVID-19 and related infection preventive measures. This agrees well with the efforts done by the Ministry of health and governmental organizations to spread the necessary information about the virus among citizens.

## Introduction

Coronaviruses are a family of Ribonucleic acid (RNA) viruses that contain positive-sense single-stranded RNA [1]. Coronavirus disease (COVID)-19 is a novel pneumonia virus that belongs to the Coronavirus family with the majority of cases by the end of December 2019 in Wuhan city located in South China [2, 3]. Coronaviruses cause respiratory, gastrointestinal, and neurological disorders. Usually, viruses affect the upper respiratory system, but they might affect the lower respiratory system in some patients. COVID-19 was declared by the World Health Organization (WHO) to be a Public Health Emergency of International Concern on March 11, 2020, as a pandemic [4]. Researchers worldwide rapidly started to isolate the novel coronavirus from confirmed infected pneumonia patients and real-time reverse transcription-polymerase chain reaction (RT-PCR) and next-generation sequencing are used to characterize it, with complete genome sequenced available on National Center for Biotechnology Information Severe Acute Respiratory Syndrome (SARS)-CoV-2 GenBank data [5, 6]. After the analysis of its genome, the Center for Disease Control and Prevention considered it a newly evolved strain of SARS that caused a pandemic back in 2002 named the new virus SARS-CoV-2 [7]. The SARS-CoV-2 virus is a beta-coronavirus, like SARS and Middle East Respiratory Syndrome, which are a large family of respiratory viruses known to cause illnesses ranging from the common cold to acute respiratory syndrome and can be transmitted *via* animal-to-human and human-to-human interaction [8]. Acute COVID-19, has a wide range of clinical manifestations, from asymptomatic infection to deadly respiratory failure, and is frequently linked with a variety of long-term complications [9]. However, elder persons and those with underlying medical problems like cardiovascular disease, diabetes, chronic respiratory disease, and cancer are more susceptible to experiencing serious illness [2].

COVID-19 spreads by person-to-person transmission among those in close contact (within around 6 feet, or 2 meters, through the respiratory droplets produced when the infected person coughs or sneezes, and direct contact and has an incubation period of about two to fourteen days [2]. On the 3^rd^ of March of 2020, the first case in Jordan was reported and the total confirmed cases are rising with reported cases discovered each day reaching a total of 512.5 million cases with around 6,3 million total death cases as of April 29^th^, 2022 [10]. At the beginning of this study, there was no antiviral treatment or vaccine available for COVID-19. Good awareness about the virus, its routes of transmission, and measures to prevent and decrease its spread might control its spread and decrease its rate of infection. Therefore, avoidance was the principal method of deterrence, and applying preventive measures to control COVID-19 infection was and still is the most critical intervention. Hence, as it says: “An ounce of prevention is better than a pound of cure”, the best way to break the circle of its transmission and prevent it from spreading is to build up a strong awareness of the COVID-19 virus, the symptoms associated with it and means of its transmission and spread. It is of great influence and an effective way to be familiar with the means of its transmission and apply it to protect yourself and others from infection such as: frequently washing hands with soap for no less than 20 seconds, or using an alcohol-based rub and avoiding touching your face. That goes well with the governmental efforts to alert the residents on the preventive measures to decrease and slow down the infection rate and the burden on hospitals during the first wave. The ministry of health along with other health organizations aimed to increase the level of awareness among citizens during the COVID-19 pandemic to decrease its spread [4, 11]. Thus, the general public knowledge, practices, and attitude to the current pandemic are critical in reducing infection rates and slowing down cases by flattening the curve during the first wave [12, 13].

Aside from the aforementioned attempts to raise public awareness, various measures and policies were implemented, including the early strict lockdown and overnight curfew in all Jordanian districts. In addition, wearing masks and gloves, and maintaining a 2-meter distance were mandatory measures in the public areas. The government prohibited traveling between cities. Certain hospitals were assigned for COVID-19 cases. COVID-19 samples were collected from residents at a reasonable cost. These measures were highly approved and implemented by the general public. All the education lectures were broadcast on radio and TV stations. Also, Jordan shifted to a distance-learning model with the help of many telecommunication companies, which provided easy and free internet access to these lectures. Mandatory PCR tests and a 14-days quarantine were provided to all the arrivals [14].

**Fig 1:**
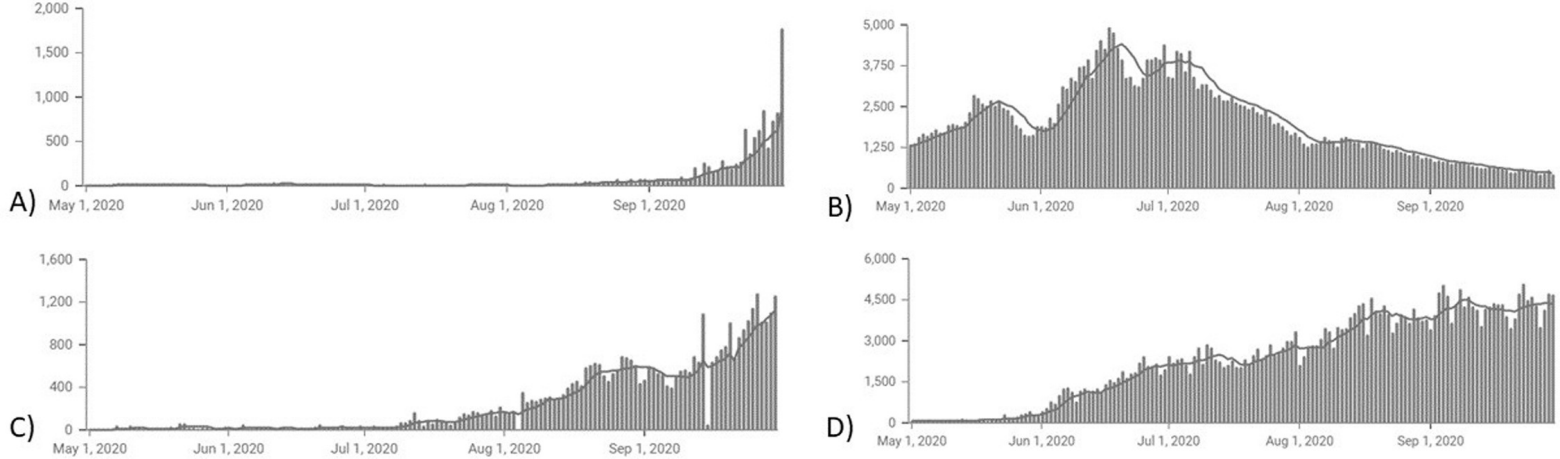
Timeline graph representing new cases reported during the period between May 1^st^, 2020 till September 30^th^, 2020. Each graph represents a country: A) Jordan, B) Saudi Arabia, C) Lebanon, and D) Iraq.

Following one of the strictest lockdowns in the world in March 2020, Jordan previously had one of the lowest infection rates of coronavirus in the Middle East. At the end of April, the mobility during the lockdown was eased and all restrictions were further removed in June. As a result of these measures, Jordan had one of the lowest infection rates of COVID-19 in the Middle East during the first wave of the disease [14]. By comparing the COVID-19 cases in Jordan with other nearby countries, we can easily observe the unique pattern of the spread of the disease (**Fig 1**).

During the time of our study, we analyzed the statistics and conducted a brief cross-country comparison with Jordan’s neighbors including Saudi Arabic, Lebanon, and Iraq. By the end of our study’s period, the number of COVID-19 cases in Jordan was 11,825 with only 57 deaths; the number of COVID-19 cases in Saudi Arabia reached 334,605 with 4,739 deaths; In Lebanon, COVID-19 cases were 39,634, and 390 deaths; And in Iraq, the cases were 362,981, while the deaths were 9,122.

It became evident in the neighboring countries the first wave began between May and June reaching a few hundred cases per day by end of July. However, in Jordan, the first wave began by end of the summer early September, much later than the neighboring. Various factors could account for such delayed spread including high restrictions and actions taken by the Jordanian government, population density, and rural areas percentages. As such, based on rural areas percentages according to World meter statistics in 2020, Jordan has the lowest percentage of 8.5% in comparison to Saudi Arabia (15.7%), Iraq (29%), and Lebanon (11%) [15]. Studies across multiple countries show higher COVID-19 susceptibility and death rates in rural areas. Finally, according to the World Health Organization, 78% of the total death in Jordan are accounted for non-communicable diseases, mainly cardiovascular diseases, cancer, diabetes, and chronic respiratory diseases [16–18].

Jordan during the first phase of the pandemic successfully maintained a low spreading rate of the COVID-19 infection, with low morbidity and mortality rate, which can be explained by the rapid governmental actions and the general public support to manage the first wave of the pandemic. The overall objective of this study is to evaluate the public knowledge and medical awareness of ways of transmission of COVID-19, and the measures of its prevention *via* a web-based designed questionnaire (i.e., online) [14].

We report in this study, the attitude, knowledge, and practice toward the COVID-19 virus within the Jordanian population. An online questionnaire was distributed randomly on different social media platforms across Jordan including 12 cities (Amman, Zarqa, Balqa, Jerash, Tafila, Irbid, Ajloon, Aqaba, Karak, Madaba, Maan, Mafraq, and Irbid). The outcomes of this research results evaluated the medical knowledge of the citizens during the COVID-19 pandemic in Jordan, which can improve the public’s awareness and practice during such pandemics and prevent its spread in the future.

## Methods

### Ethics statement

The World Medical Association Declaration of Helsinki guidance was followed in the study, which was ethically approved by the Scientific and the Ethics Committees Review Board at Zarqa University in Jordan (no. 3/2/2019-2020).

### Study design

A cross-sectional study was conducted online and distributed across Jordan’s cities that included more than eight cities, and most responses collected were from four major cities in Jordan including Amman, Zarqa, Irbid, and Mafraq during the period of May-beginning of October 2020. An online self-administered questionnaire was formatted into the Google forms and a link was generated, and randomly distributed. Jordan’s population in 2022 is currently estimated to be 10,885,459; with a life expectancy of 80.1 years. There are 3,802,228 young people under 15 years old; 6,458,558 persons between 15 and 64 years old, and 521,088 persons above 64 years old [19]. These numbers estimate that the target study population is around 6,979,646 citizens; excluding the young people under 15 years old, with an ideal sample size of 385 at a 95% confidence level [20]. The internet users in Jordan are estimated to be 66.8% in January 2021 with a total of 6.84 million internet users [21]. The survey was distributed on some social media platforms (Facebook, Whatsapp, and Microsoft apps) and was filled up completely by 328 participants from individuals at their homes or others attending universities and workplaces. Written informed consent was obtained from all subjects involved in the study. All questionnaires were filled out voluntarily and anonymously.

### Study tool

The questionnaire was developed into closed/open-ended questions and reviewed by experts within the faculty members of Pharmacy College at Zarqa University. To troubleshoot and ensure the quality of the questions and detect any possible difficulties or un-clarity during the filling, a pilot study of 30 participants from the target population was performed. Data obtained from the internal piloting study was excluded from the main study analysis. The survey questionnaire is designed in the Arabic language, as it is the official language in Jordan, and is composed of five main sections; the first section aimed to collect the socio-demographic data and lifestyle practices of the participants, which included gender, age, educational level and discipline, social status, occupation, smoking, and health status (S1 File). The second part aimed to assess the participant’s knowledge and awareness about COVID-19, including the symptoms, route of transmission, source of information, protection measures, and the high-risk groups of the viral infection. The third and fourth sections were regarding the participant’s attitude and explicitly asked about the use of supplements and natural sources to increase immunity against the virus and the actions taken if showing signs and symptoms of COVID-19. Finally, the participants were asked in the fifth section to answer questions regarding the COVID-19 testing and samples used for testing. Participants were allowed to choose more than one answer when appropriate.

### Data analysis

Data were coded and inputted into IBM Statistical Package for Social Sciences (SPSS^©^) Statistics for Windows, Version 22, (IBM Corp., Armonk, N.Y., USA), for statistical analysis. Incomplete responses were excluded from the analyses. Descriptive statistics were used to analyze the data. Differences between various groups were evaluated using the Pearson chi-Square correlation test χ² and Fisher exact tests for categorical dependent variables. *P*-values<0.05 were considered statistically significant. To measure the association between variables, the Cramer’s V coefficient test was used. Results of Cramer’s V coefficient test was interpreted as >0.25 = very strong, >0.15 = Strong, >0.10 = moderate, >0.05 = weak and >0 = no or very weak following “A guide to appropriate use of correlation coefficient in medical research” by Mukaka, 2012. Figures were plotted using Microsoft Excel 2010. Since a small number of the respondents chose to answer “I don’t know” and due to the limited space, we excluded the “I don’t know” data from the tables and figures; thus anything below a 100% response rate is due to that exclusion.

## Results

### Study sample and demographic data

Participants’ responses to the online questionnaire entitled “Evaluation of Jordan’s citizens’ awareness about the emerging Coronavirus (COVID-19) disease symptoms, its ways of transmission and the preventive measures” were considered for the study if they completed the questionnaire and approved the informed consent for participation.

The socio-demographic data is shown in **Table 1**. About 107 (32.6%) participants are males, and 221 (67.4%) are females. Among them, 48.8% and 47% were either single or married, respectively. In the present study, the majority of the participants, a total of 246 (75.0%), fell within the age group 20–40 years. With a total number of 205 (62.5%) who acquired an undergraduate university degree as their highest degree of education level, while 61 (18.6%) were high school graduates and the rest 30 (9.1%) and 32 (9.8%) had obtained a diploma and graduate degree, respectively. Regardless of the variation in education levels, and taking into consideration that the exchange rate is 1 Jordanian Dinar (JD) = 0.71 United States dollars ($), the majority of around 213 (64.9%) had a monthly income of less than 500 JD, which equals 350 $, and 74 (22.6%) with an income between 500–1000 JD (350–700 $), indicating that the minimum wage in Jordan is around 200 $. A total of 151 (45.0%) participants have jobs associated with the medical field were a total of 151 (45.0%) while the remaining did not. Around 224 (68.3%) participants were healthy whereas, 104 (31.7%) participants were reported with chronic diseases namely allergic rhinitis, diabetes mellitus, and respiratory and cardiovascular diseases. Additionally, most participants, 223 (68.0%), never smoked; while 97 (29.6%) participants were current smokers, and 8 (2.4%) participants were previous smokers and now are quit.

**Table 1:** Socio-demographic characteristics (N = 328).

	Number (%)
**Age group (years)**	
Age 15–19 years	32 (9.8)
Age 20–40 years	246 (75.0)
Age 41–55 years	31 (9.5)
Age > 55 years	19 (5.8)
**Gender**	
Male	107 (32.6)
Female	221 (67.4)
**Marital status**	
Single	160 (48.8)
Married	154 (47.0)
Divorce	8 (2.4)
Widow	6 (1.8)
**Association with the medical field**	
Yes	151 (46.0)
No	177 (54.0)
**Income JD, ($)**	
< 500 JD, (<350 $)	213 (64.9)
500–1000 JD, (350–700 $)	74 (22.6)
1001–1500 JD, (701–1065 $)	21 (6.4)
> 1500 JD, (>1065 $)	20 (6.1)
**Education level**	
High-school	61 (18.6)
Diploma	30 (9.1)
University	205 (62.5)
Graduate degree	32 (9.8)
**Smoking**	
Yes	97 (29.6)
No	223 (68.0)
Previous smoker	8 (2.4)
**Chronic disease**	
Cardiovascular diseases	16 (4.9)
Diabetes	9 (2.7)
Respiratory disease	14 (4.3)
Other chronic diseases	65 (19.8)
No	224 (68.3)

JD = Jordanian Dinar, $ = United States Dollar

### Sources of knowledge about COVID-19

**Fig 2:**
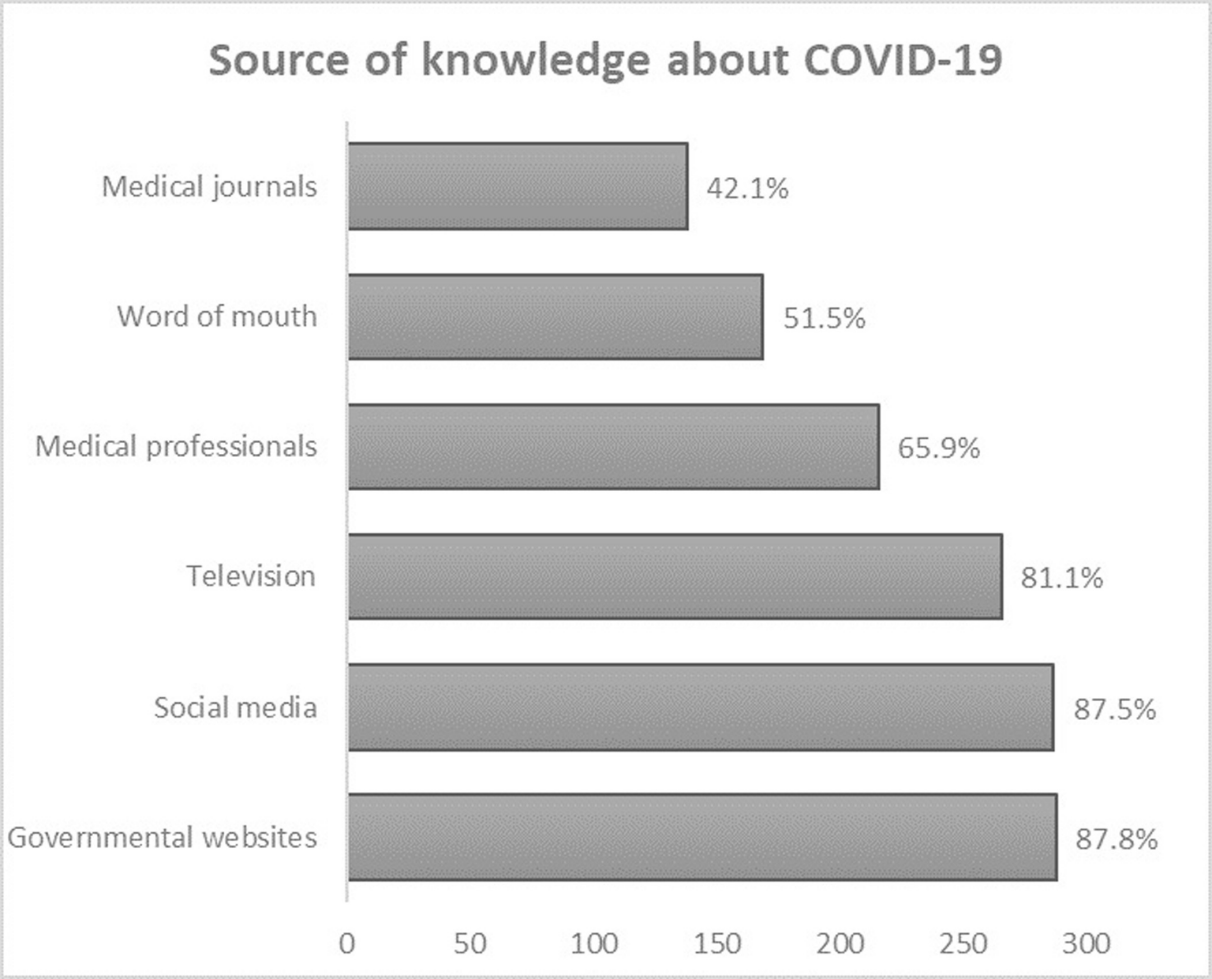
Reported source of knowledge about COVID-19.

**Fig 2** shows the various sources of knowledge and information about COVID-19 among the participants, which mainly were 87.8% the government websites, 87.5% on social media, and 81.1% on TV. Other sources of information include medical professionals (65.9%) and word of mouth (51.5%), while the least source of information was medical journals (42.1%).

### Awareness and knowledge about COVID-19

The l of knowledge among the participants about routes of transmission is shown in **Table 2**. The result displays the participant’s understanding of the possible route of transmission of COVID-19. Evidence suggests most participants view the drizzle of cough/sneeze from infected individuals (96.3%, N = 316), direct contact with contaminated surfaces (91.5%, N = 300), and the direct contact with infected individuals (84.5%, N = 277) as the main routes of the virus transmissions leading to the spread of the disease. Moreover, 45.7% (N = 150) and 43.0% (N = 141) of the participants chose the route of transmission by touching the blood of the infected person and remnants of infected individuals of the COVID virus, respectively. On the other hand, a low number of participants, ranging from 10–26%, answered “yes” to the route of transmission by shipments from China (26.8%), pets (19.2%), wild animals (16.8%), and lastly the present in the proximity of infected individuals (10.4%).

**Table 2:** Level of knowledge among the participants about routes of transmission (those who answered by choosing “yes”).

Total respondents (N = 328)		Number (%)
1	Drizzle of cough/sneeze from infected individuals	316 (96.3)
2	Direct contact of contaminated surfaces	300 (91.5)
3	Direct contact with infected individuals	277 (84.5)
4	Transmit by touching the blood of infected person	150 (45.7)
5	Remnants of infected individuals	141 (43.0)
6	Shipments from China	88 (26.8)
7	Pets	63 (19.2)
8	Wild animals	55 (16.8)
9	Present in proximity of infected individuals	34 (10.4)

**Table 3** summarizes the level of knowledge among the participants about the COVID-19 symptoms. The highest rates chosen as symptoms of the viral infections were the following: high-grade fever, troublesome breathing, coughing, headache, and loss of smell and loss of taste by 99.1% (N = 325), 96.6% (N = 317), 92.7% (N = 304), 91.2% (N = 299), 80.8% (N = 265) and 80.8% (N = 265), respectively. Moreover, ranging from 151 to 215 (46–65%) participants answered “yes” to the symptoms of sneezing, flu/cold-like symptoms, vomiting, and diarrhea. Finally, a few participants with less than 31% believed that hyperglycemia (N = 24), high blood pressure (N = 59), and the formation of blood clots (N = 104) are symptoms of the viral infection.

**Table 3:** Level of knowledge among the participants about symptoms of COVID-19 (those who answered by choosing “yes”).

Total respondents (N = 328)		Number (%)
1	High grade fever	325 (99.1)
2	Troublesome breathing	317 (96.6)
3	Cough	304 (92.7)
4	Headache	299 (91.2)
5	Loss of smell	265 (80.8)
6	Loss of taste	265 (80.8)
7	Flu/cold symptoms	215 (65.5)
8	Sneezing	211 (64.3)
9	Diarrhea	209 (63.7)
10	Vomiting	151 (46.0)
11	Formation of blood clots	104 (31.7)
12	High blood pressure	59 (18.0)
13	Hyperglycemia	24 (7.3)

The level of attitude and knowledge among participants about protection measures from COVID-19 is shown in **Table 4**. More than 92% of the participants strongly agreed on the behavioral protective measures such as no face touching, wearing a mask outside and when showing flu/cold symptoms, the use of alcoholic hand disinfectants and sanitizers, and the need for lockdown and self-isolation to protect themselves, their loved ones and the society. Most participants (N = 306, 93.3%) answered correctly that one should keep more than two meters distance rather than one meter from others to effectively protect oneself. Moreover, 90.2% (N = 296) agreed that washing hands with water and soap for at least 20 seconds rather than 10 seconds or with water only. Last, but not least, a few had a positive attitude towards the use of medications as a preventive measure including the use of paracetamol, antibiotics, and ibuprofen by 59.1%, 27.1%, and 27.1%, respectively.

**Table 4:** Attitude and knowledge about protection measures from COVID-19 (those who answered by choosing “yes”).

Total respondents (N = 328)		Number (%)
1	No face touching	323 (98.5)
2	Wearing masks outside	323 (98.5)
3	Wearing masks when showing flu/cold symptoms	323 (98.5)
4	Using alcoholic hand disinfectants/sanitizers	322 (98.2)
5	Lockdown and self-isolation measures	304 (92.7)
6	Paracetamol use	194 (59.1)
7	Antibiotics use	89 (27.1)
8	Ibuprofen use	89 (27.1)
9	Keep more than 2m distance from others	306 (93.3)
10	Keep 1m distance from others	73 (22.3)
11	Washing hands with water and soap for 20 sec	296 (90.2)
12	Washing hands with water and soap for 10 sec	70 (21.3)
13	Washing hands with water	69 (21.0)

**Fig 3:**
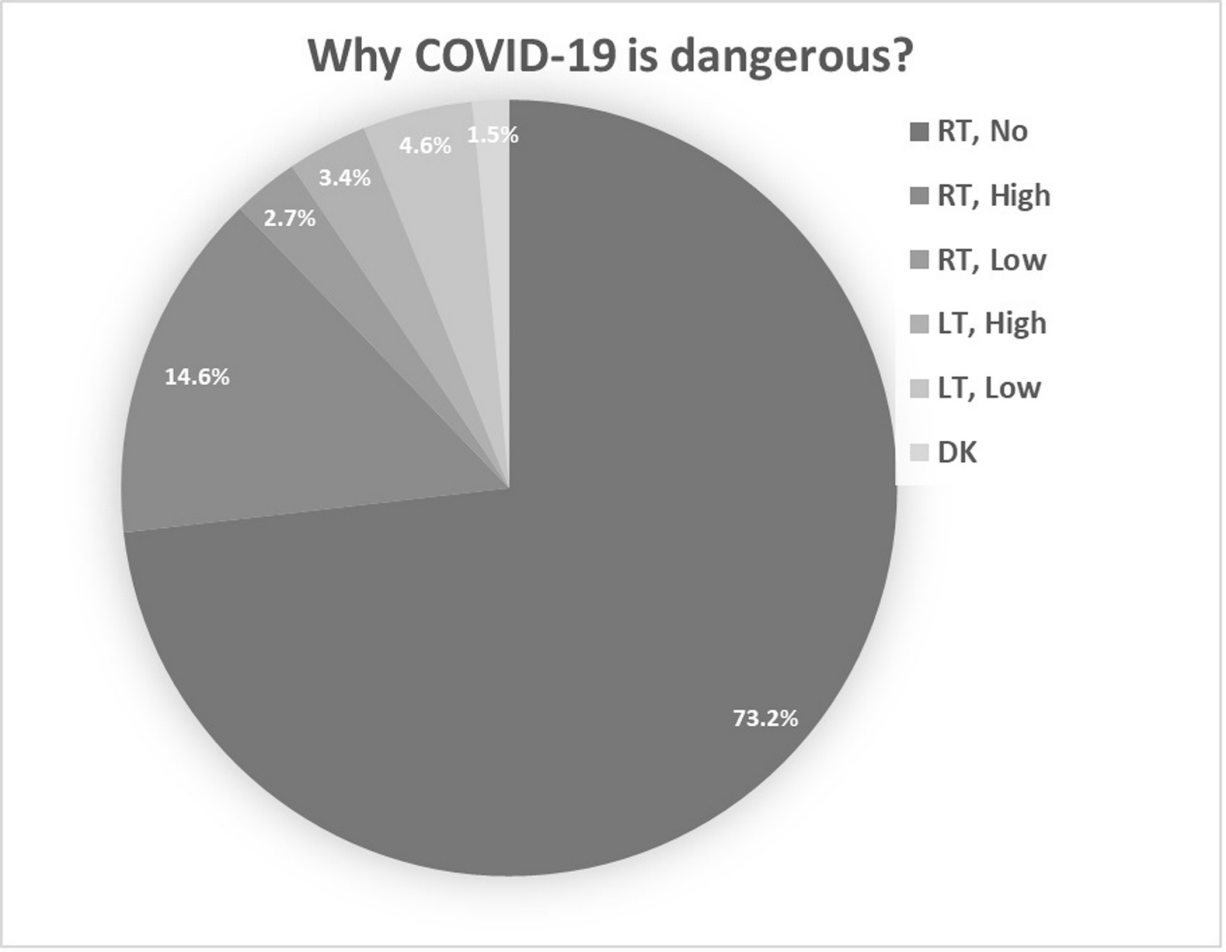
Knowledge about why COVID-19 is dangerous? (N = 328). RT, No: Rapid transmission rate with no mortality risk, RT, High: Rapid transmission rate with high mortality risk, RT, Low: Rapid transmission rate with low mortality risk, LT, High: Low transmission rate with high mortality risk, LT, Low: Low transmission rate with low mortality risk and DK: don’t know.

**Fig 4:**
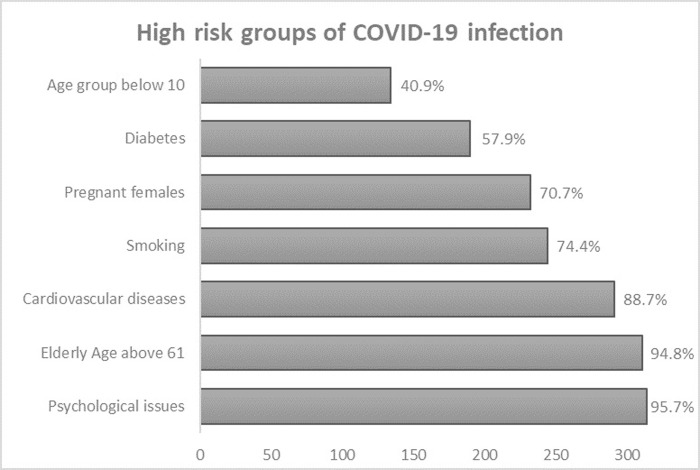
Knowledge about high-risk groups of COVID-19 infection.

**Fig 5:**
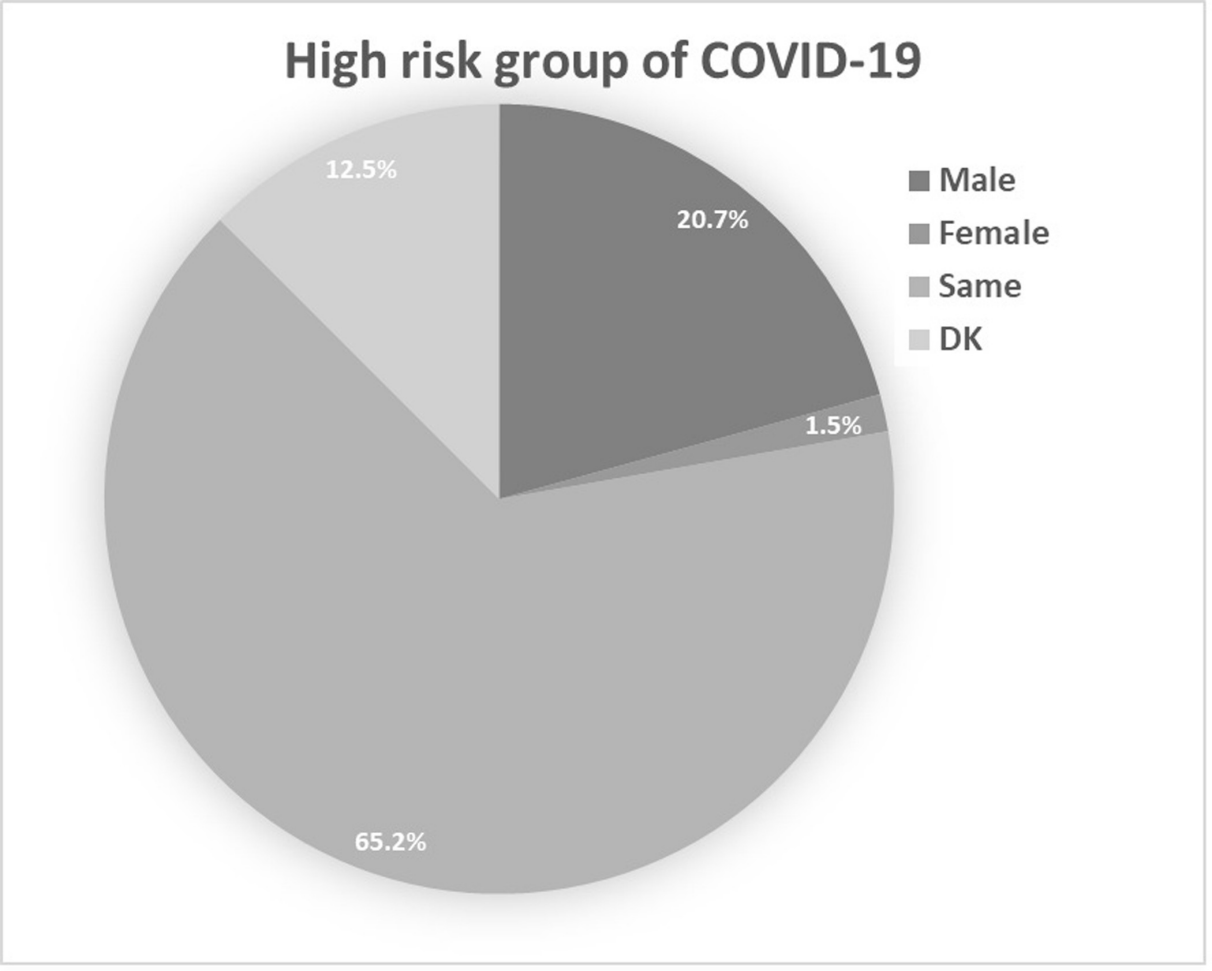
Knowledge about high-risk groups of Covid-19 infection among gender. Same: the same risk for both genders, DK: don’t know.

**Fig 3** presented the knowledge about why COVID-19 is dangerous among the participants. Rapid transmission rate with no mortality risk was the commonest view of the risk of the virus at 73.2%, followed by rapid transmission rate with high mortality risk (14.6%), low transmission rate with low mortality risk (4.6%), low transmission rate with high mortality risk (3.4%) and the lowest with a view of the virus being risky due to rapid transmission rate with low mortality risk (2.7%). A handful of 5 participants (1.5%) answered “don’t know” regarding the risk of the COVID-19 virus. Furthermore, **Fig 4** summarizes the knowledge and attitude towards the high-risk groups of COVID-19 infection. Most participants agreed that people with psychological issues, the elderly of age above 60, and people with cardiovascular diseases are at the highest risk of COVID-19 infection at 95.7%, 94.8%, and 88.7%, respectively. Additionally, 74.4% and 70.7% of the participants answered yes to people who smoke and pregnant females being at high risk of the virus, while only 57.9% viewed diabetic patients as at high risk. Only 40.9% of the participants viewed children in the age group below 10 are at high risk of COVID-19. Finally, when asked about the gender and if there is a difference in being at risk, the majority of participants (65.2%) answered the risk of COVID-19 is the same for both genders, while 20.7% viewed males being at a higher risk, 1.5% viewed female being at higher risk and the rest (12.5%) answered “don’t know” (**Fig 5**).

### Actions towards signs and symptoms of COVID-19

Lastly, **Table 5** showed the actions taken if showing signs and symptoms of COVID-19 among the participants. The results revealed that 199 participants (60.7%) would visit the physician once showing symptoms and 103 (31.4%) would not visit the physician. Once it comes to self-prescription, the majority at 55.8% choose paracetamol while an almost scarce number of participants would use self-prescribed anti-inflammatory drugs such as ibuprofen or self-prescribed zinc and vitamin c by only 5.8% and 0.6%, respectively. Thus, more than 71% of the participants answered “No” to self-prescribed ibuprofen, zinc, and vitamin C.

**Table 5:** Level of actions taken if showing signs and symptoms of COVID-19 among the participants, N = 328.

Actions taken	N	%
**Visiting the physician**		
Yes	199	60.7
No	103	31.4
DK	26	7.9
**Self-prescription Paracetamol**		
Yes	183	55.8
No	70	21.3
DK	75	22.9
**Self-prescription of anti-inflammatory drugs such as (Ibuprofen)**		
Yes	19	5.8
No	234	71.3
DK	75	22.9
**Self-prescription of zinc and vitamin c**		
Yes	2	.6
No	251	76.5
DK	75	22.9

DK: don’t know

The association between knowledge sources and socio-demographic factors is summarized in **Table 6**. A significant association was found between participants working within the medical fields and three factors. First, a significant association with the knowledge sources from medical journals (*P-value* .000**) and secondly, from medical professionals (*P-value* .000**) with participants working in a medical field, while participants working within non-medical fields had a significant association with the knowledge source from social media (*P-value* .031*). Thirdly, a strong association was also seen with obtaining facts from TV, where participants in the non-medical proficiencies had more tendency to do so. Furthermore, a significant association of *P-value* .039* was also found between the participants working within non-medical fields and the physician visit in case of COVID-19 symptoms (**Table 7**). Lastly, a significant association between participants’ income range and their beliefs about the inaccuracy of COVID-19 testing results is shown in **Table 8** (*P-value* .046*).

**Table 6:** Association between knowledge sources and socio-demographic factors.

	Facts from social media			Facts from Medical Journals			Facts from TV			Facts from medical professionals		
N (%)	Yes	No	*P-value*	Yes	No	*P-value*	Yes	No	*P-value*	Yes	No	*P-value*
**Gender**												
Male	94 (87.9)	12 (11.2)	.830	51 (47.7)	46 (43.0)	.270	88 (82.2)	14 (13.1)	.112	51 (47.7)	46 (43.0)	.419
Female	193 (87.3)	24 (10.9)		87 (39.4)	116 (52.5)		178 (80.5)	40 (18.1)		51 (47.7)	46 (43.0)	
**Association with the medical field**												
Yes	125 (82.8)	24 (15.9)	.031*	86 (57.0)	53 (35.1)	.000**	110 (72.8)	34 (22.5)	.001*	120 (79.5)	25 (16.6)	.000**
No	162 (91.5)	12 (6.8)		52 (29.4)	109 (61.6)		156 (88.1)	20 (11.3)		96 (54.2)	70 (39.5)	
**Association with Education level**												
High school	50 (82.0)	10 (16.4)	.495	22 (36.1)	32 (52.5)	.370	48 (78.7)	13 (21.3)	.380	34 (55.7)	23 (37.7)	.281
Diploma	28 (93.3)	1 (3.3)		16 (53.3)	12 (40.0)		26 (86.7)	3 (10.0)		17 (56.7)	12 (40.0)	
Bachelor university degree	182 (88.8)	21 (10.2)		82 (40.0)	107 (52.2)		163 (79.5)	35 (17.1)		140 (68.3)	54 (26.3)	
Postgraduate degree	27 (84.4)	4 (12.5)		18 (56.3)	11 (34.4)		29 (90.6)	3 (9.4)		140 (68.3)	54 (26.3)	

**P-value ≤ 0.005,

*P-value ≤ 0.05. (%) reflects % within each row.

TV = Television

**Table 7:** Association between physician’s visits and socio-demographic factors.

	Physicians visit in case of symptoms		
N (%)	Yes	No	*P-value*
**Gender**			
Male	61 (57.0)	41 (38.3)	.087
Female	138 (62.4)	62 (28.1)	
**Association with the medical field**			
Yes	83 (55.0)	58 (38.4)	.039*
No	116 (65.5)	45 (25.4)	
**Association with Education level**			
High school	40 (65.6)	14 (23.0)	.329
Diploma	19 (63.3)	11 (36.7)	
Bachelor university degree	121 (59.0)	66 (32.2)	
Postgraduate degree	19 (59.4)	12 (37.5)	

**P-value ≤ 0.005,

*P-value ≤ 0.05. (%) reflects % within each row.

**Table 8:** Association between income and beliefs about the accuracy of COVID-19 testing results.

	Beliefs about the accuracy of testing results		
N (%)	Accurate	Inaccurate	*P-value*
**Income range**			
< 500 JD, (< 350 $)	41 (19.2)	133 (62.4)	.046*
500–1000 JD, (350–700 $)	13 (17.6)	48 (64.9)	
1001–1500 JD, (701–1065 $)	2 (9.5)	10 (47.6)	
> 1500 JD (> 1065 $)	5 (25)	12 (60)	

*P-value ≤ 0.05. (%) reflects % within each row.

JD = Jordanian Dinar, $ = United States Dollar

## Discussion

Since declaring COVID-19 as a pandemic, many efforts are made by the Ministry of Health in Jordan and the government organizations, with the help of media sources, to decrease the spread of the virus. One of the best methods to halt the virus spreading is to increase the awareness of the citizens about the importance of good hygienic procedures and infection prevention practices. Thus, it is expected that Jordan’s citizens have good knowledge about the COVID-19 virus, the preventive measures to decrease the disease spreading, and the practices that should be done. This study evaluated the knowledge and medical awareness among Jordan citizens about the COVID-19 virus.

A cross-sectional study about the Jordan and Iraqi public’s awareness of the COVID-19 pandemic and the assessment of the differences between the two populations [22]. The results of our study agree well with the results collected from Jaber *et al* study as both studies showed that social media, medical staff, and official reports are the major means of information, while word of mouth and medical journals were the minors [22]. In contrast, our study revealed that TV is one of the main means of information. This difference might, first, be due to the difference in the period of collecting data and, second, can also be explained by the focus of the local media on the Minister of Health’s statements on the Jordanian National TV regarding the day to day case reports, the advice given to the public locally and internationally on how to handle the disease and the protection against COVID-19 virus. Given the fact that this agrees with the results of a study performed on the students at the University of Jordan [23]. Interestingly, the participants working within the medical fields focused on medical journals and medical professionals as the main sources of information, while participants working within non-medical fields chose social media as the main source of information.

The level of knowledge about routes of transmission was high as most of the participants (>91%) confirmed the following routes of transmission including a drizzle of cough/sneeze from infected individuals, direct contact with contaminated surfaces, and then direct contact with infected individuals. These results were much better than the previous results (80%) obtained by Jaber, *et al* as their results were earlier and this goes well with the governmental and Ministry of Health efforts to increase the citizen’s awareness [22]. On the other hand, while research has specified the possible routes of transmission for the virus [24], less than 46% knew that contamination with the remnants of the infected individuals is a possible route of transmission and didn’t know that the blood of the infected person can’t transfer the virus. This might be due to the fewer public reports about those issues and the mistaken information transmitted through the media [25].

The level of knowledge about the COVID-19 symptoms was highly accurate among the participants. The highest rates chosen as symptoms of the viral infections (>80%) were correct including high-level fever, troublesome breathing, coughing, headache, loss of smell, and loss of taste. Also, a fair percent of participants knew that sneezing, flu/cold-like symptoms, vomiting, and diarrhea are symptoms associated with COVID-19 infection. Those results confirm the improvement of knowledge about the virus over time when comparing them with Jaber *et al* study [22]. Conversely, less than 31% of participants wrongly believed that hyperglycemia, high blood pressure, and the formation of blood clots are symptoms of viral infection.

Most of the participants have good knowledge about protection measures from COVID-19 including no face touching, wearing a mask outside and when showing flu/cold symptoms, the use of alcoholic hand disinfectants and sanitizers, and the need for lockdown and self-isolation. This may be explained by the governmental organizations’ strict measures in wearing masks and cleaning hands with alcohol sanitizers, plus the Ministry of Health and social media concentration on those preventive procedures [14]. Most participants (>90%) know about keeping a distance of more than 2 m and agreed on washing hands with water and soap for at least 20 seconds to effectively protect themselves. A moderate percentage of the participants (60%) believed that they should visit the physician if they got any manifestations, while 59% chose using paracetamol as a preventive measure for COVID-19 viral infection, and only 27% chose Ibuprofen. At the beginning of this pandemic, there was a concern about using Ibuprofen since it causes an increase in the levels of angiotensin-converting enzyme-2, which is the same target of the COVID-19 virus to enter the cells. However, the data provided so far do not show any harmful effects of using Ibuprofen or NSAIDs. However, since paracetamol has lesser side effects and is safer to use, it is preferred for the management of COVID-19 accompanied fever [26]. Additionally, only 27% have a wrong perception that using antibiotics is good in treating COVID-19 viral infection. Still, it is recommended to avoid antibiotics, including azithromycin, in case of COVID-19 viral infection [27]. Additionally, a high percentage of participants (73.2%) thought that the primary danger of COVID-19 viral infection is its rapid transmission rate but the infection has no mortality risk. This might explain the rapid increase in the number of COVID-19 cases after the end of quarantine and before most Jordan citizens get vaccinated [10].

Most participants (>94%) agreed that the elderly of age above 61 are at the highest risk of COVID-19 infection, and 88.7% of participants indicated that people with cardiovascular diseases are also highly affected [28, 29]. Additionally, >70.0% of the participants agreed that people who smoke and pregnant females are also at high risk of the infection by the virus, while only 57.9% viewed diabetic patients as at high risk and these are moderate to high right responses. Those responses agree well with the studies showing that the elderly whose age is >61 years old, and people with chronic disorders such as hypertension, diabetes, cardiovascular disease, or chronic lung disease, as well as obesity and smoking were associated with increased risks for COVID-19 infection [28–30]. In addition, studies recommend pregnancy as a risk factor for COVID-19 infection as the pregnant women’s immune responses are changed during pregnancy rendering them more susceptible than non-pregnant women [31, 32].

On the other hand, 40.9% and 95.7% of the participants viewed children aged below 10 years and people with psychological issues are at high risk of COVID-19, respectively. The latter results were not true and people might be misled by the websites’ mistaken information or exaggerating the severity of the viral infection as the infection is uncommon in children less than 6 years of age and the psychiatric condition doesn’t affect the susceptibility to the viral infection [25, 33, 34]. In addition, 65.2% of the participants declared that there is no difference between genders of being at risk, while 20.7% viewed males as being at a higher risk, only 1.5% viewed females as being at higher risk and the rest (12.5%) answered: “don’t know”. However, studies show that males are more likely to be infected [28, 35, 36].

Furthermore, asking the participants about the actions taken when showing signs and symptoms of COVID-19, 60.7% revealed that they would visit the physician once showing symptoms, while 31.4% stated they would not visit the physician. Interestingly, a significant association of *P-value* .039* was found between the participants working within non-medical fields and the physician visit in case of COVID-19 symptoms, which is highly understood. While 55.8% of the participants admitted self-medication with paracetamol, few (<6%) participants would use self-prescribed anti-inflammatory drugs such as ibuprofen or self-prescribed zinc and vitamin C. Paracetamol and Ibuprofen are over-the-counter medications that are used widely for the treatment of pain and fever during COVID-19 pandemic. There were claims that Ibuprofen use during COVID-19 infection is unsafe, so people use paracetamol to decrease the pain and the fever associated with the infection. However, it was cleared by many studies that ibuprofen use was not associated with worse clinical outcomes, compared with paracetamol or no antipyretic [37, 38]. Although many studies were done on the effect of zinc and vitamin C on the COVID-19 symptoms, and some advised taking these medications to prevent or treat COVID-19 viral infection, still there is no confirmed evidence that any of these can prevent or treat COVID-19 [39–42]. Lastly, there is a significant association between increasing the participants’ income range and the beliefs of the inaccuracy of COVID-19 testing results, which might be explained by the knowledge that many could have the disease without symptoms or have the symptoms of other viral infections with the test results negative (false-negative) for COVID-19 [43].

## Limitations of the study

This study is an online study, which might affect the participants’ quality to be younger and/or highly educated with access to the social media platforms and knowledge on the use the of internet and updated technologies. Due to limited internet or social media access, the older generation is under-represented in our sample study, leading to an over-representation of females and younger age groups and limiting the responses range of perspectives and attitudes associated with pandemics.

## Conclusion

This study showed that Government websites, social media, and TV were the most common sources to get the right information related to the COVID-19 virus. The citizens have adequate information about the COVID-19 viral infection, which might be due to the efforts made by the Jordanian Ministry of Health, along with WHO and the governmental sites. Still, there is much false or uncertain information that needs more attention to be paid to get a better understanding of this viral infection, and be able to control, and stop its spreading. Hence, it is recommended that the public in such pandemics should be always educated about the advantages of following the ministry of health recommendations. Governments should spread such information through regular reports as the public consider these governmental websites a reliable source of information as demonstrated from our results to be the most used method to learn about the disease and protective measures.

## Supporting information

S1 File The form is in the original language (Arabic language). (PDF)
