# Supplementary material for: Evaluation of Jordan’s citizens’ awareness of the emerging Coronavirus (COVID-19) disease: A mixed analysis of the symptoms, transmission, and preventive measures
Source: PLOS Glob Public Health. 2022 Sep 28;2(9):e0001041. doi: 10.1371/journal.pgph.0001041 (PMC10022341; doi:10.1371/journal.pgph.0001041)
Supplement: S1 File — (PDF) [file pgph.0001041.s001.pdf]

تقييم وعي المواطنين الأردنيين بأعراض مرض فيروس كورونا المستجد (COVID-19) وطرق انتقاله والتدابير الوقائية منه

مرحبا بكم في الأستبيان حول فيروس كورونا المستجد.

يهدف هذا الأستبيان الى تقييم مدى معرفتك بأعراض فيروس كورونا المستجد ، طرق انتقاله وطرق الوقاية منه. هذا الأستبيان جزء من البحث الذي يتم إنجازه من قبل باحثين في كلية الصيدلة، جامعة الزرقاء.

إن مشاركتك في تعبئة هذه الاستبانة طوعية بشكل كامل ويمكنك الانسحاب أوالتوقف عن الاجابة في أي وقت، مع العلم أن مشاركتك في هذه الإستبانة العلمية تسهم بشكل كبير في نشر العلم وزيادة المعرفة وإثراء الخبرات العلمية والعملية، وتساعد في حل المشاكل الصحية والمجتمعية.
جميع البيانات والمعلومات التي يتم جمعها ستستخدم لغايات بحثية فقط، مع الاحتفاظ الكامل بسرية وخصوصية المشاركين فيها.

شكراً لتعاونكم .

* Required

هل توافق على المشاركة في هذا الاستبيان ؟ *

- ○ أوافق على المشاركة
- ○ لا أوافق على المشاركة

1- الفئة العمرية التي تنتمي اليها : *

- ○ 15-19
- ○ 20-40
- ○ 41-55
- ○ أكثر من 55

2-الجنس : *

- ○ ذكر
- ○ أنثى *

Pre-fill responses, then click "Get link"

3- إذا كنتِ أنثى* ، فهل أنتِ ...؟

	نعم	لا	لا أعلم
حامل	○	○	○
مرضع	○	○	○

4- الحالة الاجتماعية: *

- أعزب / عزباء
- متزوج / متزوجة
- مطلق/ مطلقة
- أرمل / أرملة

5- طبيعة العمل: *

- مهنتي صحية :طبيب، صيدلاني، ممرض، مهن طبية مساعدة وغيرها
- مهنتي غير صحية

6- مستوى الدخل: *

- أقل من 500
- من 500- 1000 دينار
- من 1001 - 1500 دينار
- أكثر من 1500 دينار

Pre-fill responses, then click "Get link"

7- مكان الإقامة *

Choose

8-ماهي أعلى شهادة حصلت عليها؟ *

- ثانوي أو أقل
- دبلوم
- جامعي
- دراسات عليا
- غير ذلك*

9-إذا كانت اجابتك على السؤال السابق غير ذلك* أذكرها.

Your answer

10-مدخن/ة *

- نعم*
- لا
- منقطع عن التدخين

Pre-fill responses, then click "Get link"

إذا كانت اجابتك على السؤال السابق (نعم*)، ما هي طرق التدخين التي تتبعها حالياً؟-11

- السجائر
- الأرجيلة
- الأرجيلة الإلكترونية
- السجائر الإلكترونية

الفترة التي إنقطعت فيها عن التدخين، في حال سبق لك التدخين-12:

- أقل من شهر
- من شهر إلى سنة
- أكثر من سنة

هل تعاني من أي من الأمراض المزمنة التالية؟-13

- أمراض قلب وشرايين
- أمراض سكري
- أمراض كلى
- أمراض كبد
- أمراض جهاز تنفسي
- غير ذلك

إذا كانت اجابتك على السؤال السابق غير ذلك* أذكرها-14.

Your answer

Pre-fill responses, then click "Get link"

الأسئلة التالية تقيس مدى معرفتك عن فيروس كورونا المستجد .

15- ما هو مصدر معلوماتك حول فيروس كورونا ؟ *

	نعم	لا	لا أعلم
وسائل التواصل الإجتماعي	○	○	○
المجلات العلمية	○	○	○
التلفاز/القنوات الفضائية	○	○	○
أناس مقربون	○	○	○
شخص يعمل في المجال الطبي	○	○	○
مواقع حكومية ورسمية	○	○	○

Pre-fill responses, then click "Get link"

16- بناءً على معرفتك، كيف ينتشر فيروس الكورونا؟ *

	نعم	لا	لا أعلم
الرذاذ المتطاير أثناء السعال أو العطاس من الشخص المصاب	○	○	○
ملامسة الشخص المصاب	○	○	○
ملامسة الأسطح المحيطة للشخص المصاب	○	○	○
التواصل عن بعد مع شخص مصاب	○	○	○
الحيوانات الأليفة	○	○	○
الحيوانات البرية	○	○	○
البضائع المشحونة من الصين	○	○	○
عن طريق فضلات شخص مصاب	○	○	○
ملامسة دم الشخص المصاب	○	○	○

Pre-fill responses, then click "Get link"

* حسب معلوماتك، ما هي أعراض الإصابة بفيروس كورونا المستجد ؟ -17

	نعم	لا	لا أعلم
السعال المتكرر	○	○	○
العطاس	○	○	○
الصداع	○	○	○
إرتفاع ضغط الدم	○	○	○
إرتفاع سكر الدم	○	○	○
ضيق التنفس	○	○	○
إرتفاع درجة حرارة الجسم	○	○	○
القيء	○	○	○
الإسهال	○	○	○
فقدان حاسة الشم و/أو التذوق	○	○	○
تجلطات في الدم	○	○	○
الرشح	○	○	○

Pre-fill responses, then click "Get link"

18- إرتفاع درجة حرارة الجسم للشخص المصاب بفيروس كورونا المستجد يكون : *

- طفيف
- متوسط
- عالي
- لا أعلم

Pre-fill responses, then click "Get link"

19- إجراءات الوقاية من فيروس كورونا المستجد تشمل: *

	نعم	لا	لا أعلم
غسل اليدين بالماء فقط	○	○	○
تعقيم اليدين بالكحول	○	○	○
الإبتعاد عن المصاب بأعراض تنفسية مسافة أقل من متر	○	○	○
الإبتعاد عن المصاب بأعراض تنفسية مسافة لا تقل عن مترين	○	○	○
تجنب لمس العينين والأنف و الفم بشكل متكرر	○	○	○
ارتداء الكمامات في الأماكن العامة	○	○	○
ارتداء الكمامات عند الاصابة بأعراض الإنفلونزا	○	○	○
غسل اليدين بالماء والصابون لمدة 10 ثواني	○	○	○
غسل اليدين بالماء والصابون لمدة 20 ثانية	○	○	○
تناول المضادات الحيوية	○	○	○
تناول البروفن كمسكن و خافض للحرارة	○	○	○
تناول الباراسيتامول كمسكن وخافض للحرارة	○	○	○

Pre-fill responses, then click "Get link"

هل تعتقد أن إجراءات العزل الذاتي وحظر التجول تساعد في وقف انتشار فيروس كورونا المستجد؟ -20 *

- نعم
- إلى حدٍ ما
- لا
- لا أعلم

طرق رفع مناعة الجسم من خلال تناول المواد الغذائية والتي تشمل: -21 *

	نعم	لا	لا أعلم
الثوم	○	○	○
الخضار الورقية	○	○	○
البروكلي	○	○	○
الحمضيات كالبرتقال والليمون وغيرها	○	○	○
تناول المكملات الغذائية من الصيدلية	○	○	○
شرب العقاقير الطبية الساخنة كاليانسون	○	○	○
شرب الماء الدافئ مع الليمون أو الخل	○	○	○
الموز	○	○	○

Pre-fill responses, then click "Get link"

22-الفئة الأكثر خطورة عند التعرض لهذا الفيروس : *

	نعم	لا	لا اعلم
النساء الحوامل	○	○	○
المصابين بأمراض القلب والكلى المزمنة	○	○	○
الفئة العمرية 10-20	○	○	○
الفئة العمرية 20-40	○	○	○
الفئة العمرية 41-60	○	○	○
الفئة العمرية 61 فما فوق	○	○	○
دون 10 أعوام	○	○	○
المدخنين	○	○	○
المصابين بالأمراض التنفسية	○	○	○
المصابين بمرض السكري	○	○	○

23- أي من التالي أكثر عرضة للإصابة بفيروس كورونا المستجد ؟ *

- ○ الذكور
- ○ الإناث
- ○ لا يوجد فرق
- ○ لا أعلم

Pre-fill responses, then click "Get link"

24- خطورة فيروس كورونا المستجد تكمن في: *

- [ ] الانتشار السريع
- [ ] الفيروس مميت وقاتل
- [ ] غير خطر والوسائل الإعلامية تبالغ
- [ ] لا أعلم

25- في حال إصابتك بأعراض رشح أو إنفلونزا ،هل سوف تقوم بزيارة الطبيب؟ *

- ○ نعم
- ○ لا
- ○ لا أعلم

26- في حال وجود حرارة أو أعراض انفلونزا بسيطة لا تستدعي زيارة الطبيب، أي من الأدوية التالية في إعتقادك تعتبر آمنة في حال الإصابة بفيروس كورونا المستجد؟ (يمكن إختيار أكثر من إجابة). *

- [ ] داكلوفيناك صوديوم مثل: فولتارين أو دايكلوجيسيك
- [ ] باراسيتامول مثل: ريفانين أو بنادول
- [ ] إيبوبروفين مثل: بروفين أو دولراز
- [ ] لا أعلم
- [ ] Other:

27- من وجهة نظرك، هل تعتقد أن فحص كورونا المستجد دقيق ؟ *

- ○ نعم
- ○ إلى حدٍ ما
- ○ لا

Pre-fill responses, then click "Get link"

28- أي العينات التالية يمكن استخدامها لإجراء فحص كورونا المستجد ؟ (يمكن اختيار أكثر من اجابة). *

- [ ] اللعاب
- [ ] المخاط
- [ ] الدم
- [ ] البول
- [ ] البراز
- [ ] لا أعلم

Get link

Never submit passwords through Google Forms.

This content is neither created nor endorsed by Google. Report Abuse - Terms of Service - Privacy Policy

Google Forms

Pre-fill responses, then click "Get link"
